# MicroRNA Markers of Previous Traumatic Brain Injury in Juvenile Offenders: Implications for Neuronal Dysfunction and Repair

**DOI:** 10.3390/genes17020134

**Published:** 2026-01-27

**Authors:** Adam T. Schmidt, Steven D. Hicks, Victoria E. Dennis, Becca K. Bergquist, Alexandra C. Bammel, Angelica Galdamez-Avila

**Affiliations:** 1 Department of Psychological Sciences, Texas Tech University, Lubbock, TX 79409, USA; victoria.e.dennis@ttu.edu (V.E.D.); bbergquist@cuyahogacounty.gov (B.K.B.); abammel@ttu.edu (A.C.B.); angaldam@ttu.edu (A.G.-A.); 2 Center of Excellence for Translational Neuroscience and Therapeutics (CTNT), Texas Tech University Health Sciences Center, Lubbock, TX 79409, USA; 3 Department of Pediatrics, Penn State College of Medicine, Hersey, Derry, PA 17033, USA; shicks1@pennstatehealth.psu.edu

**Keywords:** traumatic brain injury, microRNA, juvenile justice, justice-involved youth, juvenile offender, head injury

## Abstract

**Background/Objectives:** Justice-involved (JI) youth frequently endorse a history of traumatic brain injury (TBI). TBI, even mild TBI, can have substantial implications for long-term neurocognitive and psychosocial functioning. However, reliable, noninvasive biological indicators of chronic brain changes remain elusive. Micro-ribonucleic acids (miRNAs) are small non-coding segments of RNA that regulate a host of cellular processes. miRNAs are perturbed immediately following TBI but may continue to show changes in the chronic phase of TBI recovery. **Methods:** We investigated miRNA expression in a group of JI youth (*n* = 42, ages 12–17 [*M* = 14.42, *SD* = 1.21; 57.1% male]) with (*n* = 22) and without reported histories of TBI. **Results:** After controlling multiple comparisons, independent samples *t*-tests revealed five miRNAs (miR-425-3p, miR-30b-5p, miR-582-5p, miR-200c-3p, and miR-150-5p) were significantly different between youth with and without a history of TBI. Among these, four (miR-425-3p, miR-30b-5p, miR-582-5p, and miR-200c-3p) showed higher expression in youth with TBI history, whereas miR-150-5 showed lower expression in youth with TBI history. Three miRNAs (miR-584-5p, miR-10b-5p, and miR-30b-5p) were significantly different between youth with and without a history of loss of consciousness (LOC). MiR-584-5p was lower in youth with LOC history, whereas miR-30b-5p and miR-10b-5p were higher in youth with a history of LOC. Many of these miRNAs have been implicated in prior studies as being involved with inflammatory processes, including neuroinflammation. **Conclusions:** These results, although preliminary, provide a starting point for understanding the cellular processes related to long-term TBI outcomes within adolescents. For example, they suggest that molecular pathways involved in stress and inflammation (as well as in certain types of behavioral disorders such as substance abuse) may be implicated in long-term brain changes following TBI during development. If replicated, it may suggest future targets for pharmacological intervention.

## 1. Introduction

Numerous environmental and psychosocial correlates of juvenile offending have been identified, including poverty, peer delinquency, inconsistent parenting, child maltreatment, and neighborhood characteristics [1,2]. An additional but less well-described factor frequently linked to juvenile justice system involvement is traumatic brain injury (TBI). Adolescents who have experienced a TBI typically engage in significantly more violent and nonviolent crimes than those without a TBI history, even after controlling for demographic features [3]. Adolescents sustaining a TBI significant enough to require hospital treatment exhibit increased rates of delinquent behavior and violent and non-violent criminality [3]. Moreover, research indicates that acquired brain injuries, especially if they are recurrent, are associated with increased risks for violent behavior among children, adolescents, and adults [4] with youth experiencing a TBI demonstrating a massive increase in criminality of any kind (6.8-fold (95% 3.0–15.2)), conduct disorder diagnosis (5.7-fold (95% 2.1–15.4)), and concomitant criminal behavior and conduct disorder (18.7-fold (95% 4.3–80.1)) [3].

Self-reported rates of a prior TBI among juvenile offenders and prison populations more generally are high, ranging from 10 to 87% [3,5,6,7,8,9,10,11,12]. A meta-analysis of nine studies regarding the incidence of TBI in juvenile offenders estimated that approximately 30% of offending youth reported a prior head injury. In the five studies that included a control group, the calculated summary odds ratio was 3.37, suggesting that for juvenile offenders, the odds of endorsing a history of TBI are 3.37 times the odds of non-justice-involved youth [13]. Gordon and colleagues [14] found that approximately 22% of adolescents in state custody in Texas reported a history of a TBI (with approximately 44% of these classified as moderate or severe), whereas upwards of 41% of juveniles in county custody who were screened reported a TBI history (with 18.5% classified as moderate or severe). This study found that, in both samples, TBI occurred prior to the age of first offense in 56.5% (state) to 78.5% (county) of juveniles. Moreover, results demonstrated that juveniles sustaining a TBI prior to their first offense committed more violent offenses and exhibited more mental health diagnoses when compared to their peers sustaining a TBI following their first offense [14]. These rates are significantly higher than those occurring within the general adolescent population and suggest that TBI may be a significant contributor to or corollary of juvenile delinquency [3,13]. Of potentially more concern, other research suggests that many justice-involved youth sustaining TBIs do not seek medical treatment or do not come to the attention of medical personnel unless their injuries are sustained when they are in custody [12]. As such, there are a large number of juvenile offenders with a history of TBI, potentially resulting in chronic TBI symptoms, who never seek or receive treatment. The implications of these issues for justice-involved youth and professionals working with these individuals may be profound.

Research associates TBI (even mild TBI) with changes to brain structure and function, including white matter microstructure [15,16,17]; sleep disorders [18]; impaired cognitive and school functioning [19,20,21,22]; substance use [23]; and psychiatric disorders, including increases in suicidal ideation [23,24,25]. In addition, TBI recovery can be greatly influenced by a child’s psychosocial environment, with children with higher levels of family dysfunction demonstrating worse outcomes [17,26]. Given the increased prevalence of TBI within justice-involved youth, the potential long-term consequences of a TBI occurring in childhood or adolescence, and the significant psychosocial stressors associated with juvenile justice involvement, many of which may also exacerbate recovery from TBI—there is a critical need to identify brain-related pathways that may undergird long-term outcomes following TBI among justice-involved youth.

Few studies of human participants, and no studies with which we are aware among justice-involved youth, have explored brain-related molecular markers of neuronal function following a TBI. This represents a gap in our understanding of how TBI may interact with other genetic or environmental vulnerabilities to impact brain, cognitive, and behavioral development and to identify potential markers of long-term dysfunction and/or targets of intervention. One reason for this gap is the difficulty in sampling molecular indicators of brain function in live human participants. However, recent advances in molecular genetics sampling and analysis make it possible to obtain markers of cellular processes underlying brain function from neuroexosomes available in saliva [27,28,29]. Specifically, micro-ribonucleic acids (miRNAs) are small, non-coding segments of RNA between 20 and 25 nucleotides in length, demonstrated to be potent epigenetic regulators of gene expression and windows into the cellular processes unfolding within the brain, including after a TBI [30]. These molecules regulate gene expression by binding to and suppressing the function of messenger RNA [30]. miRNAs regulate biological processes associated with TBI recovery and pathophysiology, including neuroinflammation, homeostasis, and apoptosis [31,32,33]. Research associates salivary miRNA alterations with mild, moderate, and severe TBI and with both the acute and chronic stages of recovery [27,28,34,35,36,37]. However, no studies have examined miRNA expression within a high-risk population or within the chronic timeframe of TBI recovery to determine if these molecules provide viable indicators of TBI recovery over an extended time and in a population with multiple risks. Given the multiple cellular processes associated with miRNA expression and the relatively non-invasive way these molecules can be obtained (i.e., through salivary samples), miRNAs hold considerable promise as potential markers of chronic brain dysfunction following TBI, potentially even into the chronic phase of recovery [28,32]. Further, they hold promise for potentially understanding how TBI may impact individuals during a dynamic phase of brain development (i.e., adolescence). Salivary miRNA samples have been shown to reflect TBI severity and to accurately index miRNA collected from the cerebrospinal fluid [27]. Further, salivary miRNA is sensitive to mild TBIs [37] and can be traced to neuroexosomes, further highlighting its ability to index brain function [27]. In a recent pilot investigation [38], we found that expression of several miRNAs related to neuroinflammation and stress exposure was related to the number of adverse childhood experiences endorsed by adolescents. Hicks and colleagues [28] also found evidence that the expression of two miRNAs (specifically, miR-28-3p and miR-339-5p) was associated with repeat concussive injuries within an adolescent sample. Taken together, these initial findings highlight the potential for miRNA to serve as markers of brain-related dysfunction within the chronic phase of TBI recovery and the potential utility of examining miRNA expression within a vulnerable adolescent sample.

The current preliminary investigation aimed to evaluate miRNA expression obtained via salivary collection in a group of justice-involved youth with a history of TBI. The specific study objectives were to determine if there were differences in miRNA expression between youth reporting a history of TBI and youth without a reported TBI history and to determine if factors such as loss of consciousness affected the pattern of miRNA expression within the TBI group. We hypothesized that miRNAs involved in brain-related processes such as neuroinflammation and stress regulation would be altered in youth reporting a prior TBI. To increase the external validity and maximize the potential utility of our findings for understanding the various presentations of TBI in justice-involved youth, youth self-reports (without corresponding medical documentation, which rarely exists in this population) were used to assess for the presence or absence of TBI, and no corrections for time since injury or mechanism of injury were undertaken.

## 2. Materials and Methods

### 2.1. Participants

For a more detailed description of experimental methods and samples, see Dennis et al., 2025, Schmidt et al., 2024 [19,38]. Briefly, data were obtained as part of a larger study on neurocognitive functioning and environmental exposures within justice-involved youth. Our sample size consisted of 42 justice-involved adolescents. Of these participants, 23 (54.76%) endorsed a history of any TBI occurrence, while the remainder did not (see Table 1 for a full breakdown of participant demographics). Participants were ineligible for the current investigation if they could not participate in neuropsychological testing (e.g., because of physical disabilities precluding standardized testing procedures or serious psychopathology such as active psychosis). Participants were compensated for their time by earning community service hours towards their probation.

### 2.2. Procedures

All study procedures were approved by the institutional review boards of the participating organizations, and all study procedures were in accordance with the American Psychological Association’s Ethical Principles and Code of Conduct as well as with international ethical standards of research involving human subjects. Participants were recruited from a juvenile justice facility in a small urban county in Northwest Texas. Recruitment occurred in two ways: (1) Participants and their caregivers were offered participation in the study in addition to other community service options as presented by their juvenile probation officers. (2) Trained graduate researchers approached eligible families at Juvenile Court. No testing occurred until consent from caregivers and assent from all youth participants was obtained, typically at the time of initial contact or, in some cases, just prior to the start of data collection. All procedures were explained to the youth and their caregivers in language that had been approved as appropriate for their educational level. Youth and their caregivers were provided the opportunity to ask any questions about the study and were informed about limits to confidentiality (i.e., that the research team would need to report any suicidal intent or plans and would need to report any information regarding abuse or neglect of a child or vulnerable adult). Data collection took place in private rooms at the juvenile justice facility or within the university research laboratory of the first author. De-identified data were stored securely in a locked file cabinet in the first author’s research laboratory, and consent and assent forms containing participant identifiers were stored separately in the locked university office of the principal investigator. Only the members of the research team (e.g., principal investigator and graduate research assistants) had access to the raw data; all raw data were de-identified, coded, and stored using participant ID numbers only, and, after data were obtained, only the principal investigator had access to the consent and assent forms that contained participant names. Participants were provided with instructions before completing each self-report measure and were told that they could halt their participation in the study at any time and that they could elect not to answer any question. Participants did not consume food or beverages for at least 30 min prior to providing the salivary sample. Participants were compensated for their time by earning community service hours at a rate of two times the hours of participation (e.g., a participant could earn up to 7 h of community service for 3.5 h of participation). Because of the nature of our sample, members of the research team directly input a youth’s community service hours into the county tracking system so that a youth’s probation officer, mental health treatment provider, or other facility staff would not be able to identify if a youth had or had not participated in the study. Because all stored data were de-identified, there was no way of identifying participants after the fact, thereby precluding probation officers/juvenile justice staff from attempting to access data about any particular youth.

### 2.3. Measures

#### 2.3.1. Demographics

Caregivers completed general demographic information (when known) regarding the youth’s biological parents (e.g., age, gender, and race), living situation, education, employment, and legal involvement. The caregiver also reported on the youth’s basic information (e.g., age, gender, and race), physical and mental health, and education.

#### 2.3.2. Traumatic Brain Injury

The Ohio State University Traumatic Brain Injury Identification Method (OSU TBI-ID) was used to capture traumatic brain injury [39]. This is a self-report of traumatic brain injury occurrence. The OSU TBI-ID is a structured self-report interview designed to systematically document exposure to head injuries by collecting detailed information on the number and timing of events, associated acute symptoms (e.g., loss of consciousness and altered mental status), and subsequent functional consequences. The measure has been widely implemented in clinical and research settings to estimate lifetime TBI severity and characterize the potential cognitive, emotional, and physical sequelae of injury. Psychometric evaluations have demonstrated strong reliability and validity evidence for the OSU TBI-ID. Corrigan and Bogner [39] reported substantial interrater reliability and predictive validity for indices derived from the interview in treatment-seeking adults. Bogner and Corrigan [40] further supported the measure’s utility in justice-involved samples, showing that OSU TBI-ID summary indices predicted relevant cognitive, affective, and behavioral outcomes. More recently, Corrigan and colleagues [41] found that self-administered versions produce patterns of classification that are largely concordant with interviewer-administered formats, suggesting that psychometric integrity is retained across administration modalities. Collectively, these findings indicate that the OSU TBI-ID offers a comprehensive and empirically supported method for identifying lifetime TBI, which is particularly critical in populations where mild or remote injuries are frequently underreported.

#### 2.3.3. miRNA Expression

For more details on miRNA expression and analysis, please see Hicks et al., 2018, 2020; Schmidt et al., 2024; and Sullivan et al., 2022 [27,28,29,38]. Briefly, salivary samples for miRNA analysis were obtained through active expectoration of 1 mL using the CP-190 nucleic acid stabilization kit (DNA Genetic; Ottawa, ON, Canada). All samples were collected with Oragene RNA stabilizer. The expression of miRNA collected via expectoration sampling is a valid approach for collecting salivary miRNA (see Sullivan et al., 2022 [29]) and is somewhat easier for researchers and research participants when compared to salivary swabbing. Moreover, all youth in the current study were sampled using expectoration methods, thereby eliminating any potential variance due to sampling method [29]. All samples were collected and stored in accordance with the manufacturer’s instructions. Saliva samples were stored at room temperature in a secure laboratory facility of the principal investigator prior to being shipped for analysis. Salivary samples were collected between September 2019 and May 2021. Most data collection occurred in the afternoon or early evening; however, the time of data collection was not strictly controlled. Prior studies suggest that the time of salivary data collection does not substantially impact miRNA expression from salivary samples [29]. Samples were stored for between one and three months prior to shipment for analysis. Samples were stored at −80 °C within twelve weeks of collection and underwent only one freeze–thaw cycle prior to RNA extraction. RNA sequencing occurred at the SUNY Upstate Molecular Core Facility (Syracuse, NY, USA). Salivary RNA was extracted using the Oragene RNA purification protocol and the RNeasy mini column (Qiagen, Germantown, MD, USA). The yield and quality of the RNA samples were assessed using the Agilent Bioanalyzer. Following library construction, multiplexed samples were run on an Illumina HiSeq instrument at a targeted read depth of 10 million reads per sample (Illumina, San Diego, CA, USA). Reads were aligned to the human genome in Partek Flow using the Bowtie 2 algorithm. Mature miRNA levels within each sample were quantified using miRBase 22. The miRNAs with raw counts <10 in >10% of samples were excluded. A Deseq normalization technique with sum scaling was applied.

We also evaluated the physiologic relevance of miRNAs that displayed relationships with self-reported TBI; target pathways were determined in DIANA miRPath v3 software [42]. Moderate interactions (microT-cds score > 0.80) between miRNA/mRNA pairs were identified, and the Kyoto Genes and Genomes (KEGG) pathways with mRNA representation exceeding that expected by chance alone were defined using Fisher’s exact test (adjusted *p* < 0.05). Relationships between miRNAs of interest and their target KEGG pathways were visualized using a heatmap with hierarchical clustering.

### 2.4. Data Analysis Plan

All analyses were conducted with SPSS v. 31.0. Because this was a pilot investigation and we did not have any established correlations or other preexisting data with which to estimate a sample size calculation, all participants with complete data were included in the analyses. Associations between miRNA and TBI occurrence were assessed with Pearson correlation coefficients. Findings with *p*-values of less than 0.05 were considered statistically significant. Next, miRNAs that were statistically significantly correlated with TBI occurrence were retained for further evaluation. To account for multiple comparisons, a Benjamini–Hochberg [43] false discovery rate (FDR) correction procedure was used. This approach adjusts for thresholds for statistical significance (starting with the nominal *p* < 0.05) for each inferential test as a function of the number of tests in a set of analyses and *p*-value rank. This approach determines the adjusted *p*-value thresholds with d × (i/n) for each original *p*-value, where d = 0.05 (the false discovery rate), i = *p*-value rank (the lowest observed *p*-value would have a rank of 1), and n = number of tests.

## 3. Results

### Findings

Results indicated that participants reporting a history of sustaining a TBI demonstrated alterations in the expression of miR-30b-5p, miR-200c-3p, miR-582-5p, miR-953p, miR-200b-5p, miR-425-3p, miR-150-5p, miR-331-3p, miR-1260a, and miR-18a-5p. Following the FDR correction, five miRNAs remained statistically significant with an adjusted *p*-value of ≤0.025: miR-425-3p (*p* = 0.004), miR-30b-5p (*p* = 0.011), miR-150-5p (*p* = 0.011), miR-582-5p (*p* = 0.017), and miR-200c-4p (*p* = 0.025). Four of the five significant miRNAs exhibited higher expression in youth with a history of TBI occurrence relative to youth without any TBI occurrence. These were miR-425-3p (mean difference = +0.227), miR-30b-5p (+0.165), miR-582-5p (+0.173), and miR-200c-3p (+0.052). In contrast, miR-150-5p demonstrated lower expression in TBI+ youth compared to TBI− youth (mean difference = −0.172).

Three miRNAs (miR-584-5p, miR-10b-5p, and miR-30b-5p) were significantly different between youth with (n = 14, 33.33%) and without a history of loss of consciousness (LOC) following FDR correction. MiR-5845p expression was lower in youth with LOC history (mean difference = −0.101), whereas miR-30b5p (mean difference = +0.139) and miR-10b5p (mean difference = +0.027) expression was higher in youth with LOC. No significant differences in correlated miRNA expression emerged between youth with no history of TBI and youth with two or more (n = 11, 26.19%). See Table 2 for descriptive statistics for the present study’s statistically significant miRNA and study-referenced TBI-associated miRNAs. 

**Figure 1:**
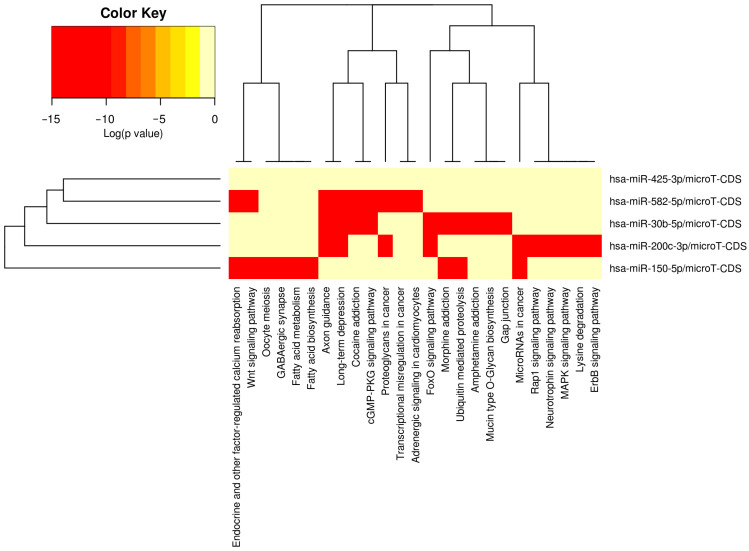
Heatmap depicting associations between miRNAs and KEGG pathways.

A total of 1158 putative gene targets were identified for the five miRNAs correlated with the presence of a TBI. KEGG is a curated database that organizes genes into biologically meaningful pathways representing molecular interactions, cellular processes, and disease mechanisms. KEGG pathway overrepresentation analyses identify pathways that are statistically enriched among a given set of genes, suggesting that these genes may converge on shared biological functions. There were 15 KEGG pathways over-represented by these gene targets (see Table 3). Brain-related pathways included cocaine addiction (hsa05030, *p* = 1.4 × 10^−5^, 10 genes targeted by 4/5 miRNAs), long-term depression (hsa04730, *p* = 0.0026, 7 genes targeted by 3/5 miRNAs), morphine addiction (hsa-5-32, *p* = 0.0027, 8 genes targeted by 4/5 miRNAs), axon guidance (hsa04360, *p* = 0.0041, 20 genes targeted by 3/5 miRNAs), dopaminergic synapse (hsa04728, *p* = 0.0092, 19 genes targeted by 4/5 miRNAs), and neurotrophin signaling (hsa04722, *p* = 0.016, 17 genes targeted by 4/5 miRNAs). Among these brain-related pathways, axon guidance and long-term depression were most highly related to miR-200c-3p, miR-30b-5p, and miR-582-5p, whereas miR-150-5p and miR-30b-5p displayed strong associations with morphine addiction, and miR-425-3p was not highly related to any brain-related pathways (see Figure 1 for heatmap).

## 4. Discussion

The current preliminary study examined the relations between microRNA expression and a history of prior self-reported TBI within a high-risk adolescent sample. Results indicated that five miRNAs were significantly related to a self-reported history of TBI, including miR-150-5p, miR-425-3p, miR-582-5p, miR-200c-3p, and miR-30b-5p. Additionally, miR-10b-5p, miR-584-5p, and 30b-5p were also different between individuals with and without a self-reported history of LOC. Many of these miRNAs have been shown to attenuate neuroinflammation (e.g., miR-30b-5p [46] and miR-582-5p [47]), suggesting a potential protective role for these molecules, whereas others have been specifically identified as potentially proinflammatory (e.g., miR-200c-3p) [48]. Moreover, microRNA miR-150-5p and miR-10b-5p have been demonstrated to be possible markers of diverse neurological diseases [49,50,51], and miR-425.3p may show changes following treatment with antidepressant medication [52]. Taken together, the current findings, in light of prior research, clearly suggest that many of the miRNAs identified in the present study index brain functions and may be viable markers of brain dysfunction/neurologic injury if validated by additional research. Moreover, although speculative given the preliminary nature of the present results, our findings suggest that the long-term trajectory of TBI recovery may be marked by an interplay of various miRNA regulators, some potentially serving to decrease inflammation, while others may be markers of proinflammatory processes.

Most of these miRNAs were also related to specific brain-related pathways implicated in substance use (miR-150-5p and miR-30b-5p) and/or to neuronal development processes (miR-200c-3p, miR-30b-5p, and miR-582-5p). It is interesting that miRNAs related to substance abuse were implicated in these findings. Justice-involved youth have high rates of substance use disorders [2], and it is plausible that exposure to a TBI during the developmental period further enhanced these risks. Unfortunately, we do not have information specific to substance use disorders in the current sample, so we were unable to evaluate if there were group differences in substance use among TBI and non-TBI participants. This is a limitation of the current findings, but it represents an important area to explore in future studies. It is also possible that sustaining a TBI may alter some of the same brain/genetic pathways implicated in substance use (e.g., gene targets involved in impulsivity, sensation seeking, and externalizing behaviors). Consistent with this possibility, prior research demonstrates behavioral overlaps and comorbidities between TBI and externalizing behaviors and substance use [21,23]. Thus, the observed pattern of miRNA expression among the TBI group may merely be a consequence of phenotypic overlap between the behavioral outcomes of chronic-phase TBI recovery and externalizing psychopathology, such as substance use disorders.

Somewhat unexpectedly, other miRNAs implicated in cumulative TBI post-recovery processes (e.g., miR-28-3p and miR-339-5p) were not found to be significantly related to a history of TBI within this sample. Moreover, the miRNAs identified in the current study also do not substantially overlap with other microRNAs indicated in other studies of TBI (e.g., miR-26b-5p and miR-221-3p) [44]. There are several potential reasons to explain the different pattern of correlations between miRNA and TBI observed in the present investigation. First, the Hicks and colleagues [28] investigation, which is the only prior study with which we are aware to specifically examine the impact of multiple prior TBIs on miRNA expression, used a sample of adult professional athletes with confirmed medical histories of multiple concussive injuries. Our current sample comprised adolescents with varying degrees of TBI exposures ranging from 1 to upwards of three or more. As such, these results may be related more broadly to TBI vs. no-TBI or to TBI occurring in the developmental period rather than specifically to the experience of cumulative concussions occurring mainly in adulthood. Second, the current findings cover a wide range of TBI severity, spanning possible mild concussions to more moderate/severe injuries resulting in LOC. As such, our sample is a mixed sample that may not represent any specific subgroup of TBI survivors but may reflect more general brain-related processes that can occur following a closed head injury. Likewise, our sample varied significantly in terms of the length of time between data collection and when an injury had occurred. As such, our current pattern of findings may reflect chronic disruption to cellular processes occurring months, if not years, following when an injury was sustained. Unfortunately, due to our small sample size and the difficulties with retrospective recall regarding TBI history, we were limited in the extent to which we could account for these variations in our sample. Finally, many prior studies using miRNA markers typically involve the acute or subacute period of time after injury, often, but not exclusively, use miRNA obtained from blood, and use less diverse samples. Therefore, it is likely that one or all of these differences explain why our findings may differ from other prior studies. Nonetheless, the results suggest that miRNA markers may continue to be sensitive to the long-term impacts of sustaining even a mild TBI far into the future. However, additional longitudinal investigations that take into account the mechanism of injury, time since injury, age at first injury, etc., and that are better powered are needed to confirm and extend these preliminary results.

It is also possible that our results reflect the unique demographic and environmental characteristics of our sample. That is, the current results are most similar to our previous investigation, which examined the relations between microRNA expression and exposure to adverse childhood experiences (ACEs) [38]. Two of the miRNAs identified in the current investigation (miR-30b-5p and miR-582-5p) were also found to be associated with the number of ACEs the youth had experienced. These microRNAs are implicated in stress regulation and inflammation, suggesting that they may remain altered/be sensitive to even small perturbations in brain function resulting from insults occurring during development (e.g., child maltreatment or mild TBI). Relatedly, it may be that our findings merely reflect miRNA markers of general stress exposure (from any cause) and may not be specific to chronic TBI or child adversity. Because there were no significant demographic differences between our TBI and control participants, we do not believe this is a likely explanation, but if future studies find similar patterns resulting from various stressors, the current findings would still have merit, as they suggest that individuals sustaining a TBI, even in the distant post-injury phase, continue to exhibit changes in brain-related miRNA expression even in the context of other stressful environmental and psychosocial influences.

The current study has multiple limitations, including a relatively small sample size; reliance on self-reports for TBI diagnosis; substantial variability of our sample, including different degrees of injury severity, injury mechanism, and time since injury; and a sample that has multiple environmental and psychosocial risk factors. Future studies should endeavor to capture as many of these demographic differences as possible across a larger population in order to untangle these various potentially contributory factors. Moreover, our findings need to be verified with larger samples that can allow for more rigorous tests of miRNA expression (e.g., breaking the sample down into training and test sets and/or using machine learning algorithms to further probe the findings). We also used expectoration sampling methods, and future research should evaluate if similar results are obtained with other miRNA collection techniques, such as salivary swab samples and/or blood miRNA collection. Finally, future studies that examine miRNA expression in relation to other cognitive/behavioral sequelae of TBI would be valuable to support the clinical utility of the current results. Given these limitations, the current results should be viewed as preliminary and, as is the case for many prior investigations of miRNA expression, need to be rigorously evaluated in carefully designed, large, longitudinal, multisite trials. Nonetheless, despite these limitations, the current pattern of preliminary findings tentatively suggests that miRNA may, in fact, be viable markers to index brain processes such as a long-term response to inflammation in adolescents with self-reported histories of TBI. If validated by future investigations, miRNA expression may lead to therapeutic innovations that could counter some of the chronic effects of TBI that occur in some populations, especially children and adolescents with significant psychosocial risks such as justice-involved youth [1,26].

## Figures and Tables

**Table 1:** Sample demographic information.

	No TBI		TBI		All Adolescents	
	*n*	Percentage	*n*	Percentage	*n*	Percentage
All Adolescents	19	45.2%	23	54.8%	42	100.0%
Age						
12- to 13-year-olds	3	7.1%	8	19.0%	11	26.2%
14- to 15-year-olds	11	26.2%	10	23.8%	21	50.0%
16- to 17-year-olds	5	11.9%	5	11.9%	10	23.8%
Mean (SD)	14.68 (1.16)		14.22 (1.24)		14.43 (1.21)	
Sex						
Male	10	23.8%	14	33.3%	24	57.1%
Female	9	21.4%	9	21.4%	18	42.9%
Race/Ethnicity						
White	5	11.9%	7	16.7%	12	28.6%
Black	6	14.3%	6	14.3%	12	28.6%
Hispanic/Latino	8	19.0%	9	21.4%	17	40.5%
Other	0	0.0%	1	2.4%	1	2.4%
Worst TBI Severity						
Mild	-		15	65.2%	-	
Moderate or Severe	-		4	17.4%	-	
Missing/Not Reported	-		4	17.4%	-	
Proportion with 2+ TBIs	-		11	47.8%	-	

Note. Percentages for the TBI-related variables (worst TBI severity and proportion with 2+ TBIs) reflect proportions of the TBI group only rather than proportions of the total sample. Significance testing for between-group differences was not conducted due to small group sizes and sparse cell counts.

**Table 2:** Descriptive statistics for the present study’s statistically significant miRNA and study-referenced TBI-associated miRNAs.

	*N*	Min	Max	Mean	*SD*
miR-30b-5p	42	−0.285	0.715	0.004	0.213
miR-200c-3p	42	−0.202	0.105	−0.104	0.076
miR-582-5p	42	−0.271	0.729	0.093	0.24
miR-95-3p	42	−0.368	0.542	−0.198	0.169
miR-200b-5p	42	−0.315	0.615	−0.124	0.183
miR-425-3p	42	−0.288	0.692	0.119	0.264
miR-150-5p	42	−0.103	0.895	0.074	0.222
miR-331-3p	42	−0.223	0.777	−0.033	0.214
miR-1260a	42	−0.265	0.653	−0.102	0.183
miR-18a-5p	42	−0.357	0.634	−0.024	0.223
miR-584-5p	42	−0.27	0.179	−0.1	0.119
miR-10b-5p	42	−0.071	0.137	−0.039	0.038
miR-28-3p *	42	−0.429	0.571	0.056	0.208
miR-339-5p *	42	−0.05	0.944	0.037	0.178
miR-221-3p **	42	−0.32	0.68	−0.077	0.200
miR-26b-5p **	42	−0.46	0.54	0.042	0.289
miR-769-5p **	42	−0.14	0.835	0.094	0.218
miR-1307-3p **	42	−0.197	0.803	0.033	0.241

Note: ** miRNA previously linked to TBI in Di Pietro and colleagues; Hicks and colleagues [44]. * miRNA previously linked to TBI in Hicks and colleagues [45].

**Table 3:** KEGG pathways over-represented by these gene targets identified for the five miRNAs correlated with the presence of a TBI.

KEGG Pathway	*p*-Value	Number of Genes	Number of miRNAs
Mucin type O-glycan biosynthesis	5.97 × 10^−7^	4	1
Cocaine addiction	1.41 × 10^−5^	10	4
Glycosphingolipid biosynthesis—lacto and neolacto series	0.0012	5	4
Gap junction	0.0026	9	3
Long-term depression	0.0026	7	3
Amphetamine addiction	0.0026	10	4
Morphine addiction	0.0028	8	4
Lysine degradation	0.0031	6	4
Adrenergic signaling in cardiomyocytes	0.0041	18	3
Axon guidance	0.0041	20	3
Dopaminergic synapse	0.0092	19	4
Transcriptional misregulation in cancer	0.0138	12	4
Neurotrophin signaling pathway	0.0165	17	4
Glycosaminoglycan biosynthesis—keratan sulfate	0.0303	3	2
cAMP signaling pathway	0.0382	23	4

## Data Availability

The raw/processed data required to reproduce the above findings cannot be shared at this time, as the data also forms part of an ongoing study.

## Abbreviations

The following abbreviations are used in this manuscript:

miRNA	Micro-ribonucleic acid
TBI	Traumatic brain injury
DNA	Deoxyribonucleic acid
USA	United States of America
ACEs	Adverse childhood experiences
LOC	Loss of consciousness
OSU-TBI	Ohio State University Traumatic Brain Injury Identification Method
